# Effect of a Nurse-Led Diabetes Self-Management Education Program on Glycosylated Hemoglobin among Adults with Type 2 Diabetes

**DOI:** 10.1155/2018/4930157

**Published:** 2018-07-08

**Authors:** Golnaz Azami, Kim Lam Soh, Shariff Ghazali Sazlina, Md. Said Salmiah, Sanaz Aazami, Mosayeb Mozafari, Hamid Taghinejad

**Affiliations:** ^1^Department of Nursing and Rehabilitations, Faculty of Medicine and Health Sciences, University Putra Malaysia (UPM), 43400 Serdang, Selangor, Malaysia; ^2^Department of Nursing, Faculty of Nursing and Midwifery, Ilam University of Medical Sciences, Ilam 693917714, Iran; ^3^Department of Family Medicine, Faculty of Medicine and Health Sciences, University Putra Malaysia (UPM), 43400 Serdang, Selangor, Malaysia; ^4^Department of Community Health, Faculty of Medicine and Health Sciences, University Putra Malaysia (UPM), 43400 Serdang, Selangor, Malaysia

## Abstract

In recent years, great emphasis has been placed on the role of nonpharmacological self-management in the care of patients with diabetes. Studies have reported that nurses, compared to other healthcare professionals, are more likely to promote preventive healthcare seeking behaviors. The aim of this study was to investigate the effectiveness of a nurse-led diabetes self-management education on glycosylated hemoglobin. A two-arm parallel-group randomized controlled trial with the blinded outcome assessors was designed. One hundred forty-two adults with type 2 diabetes were randomized to receive either usual diabetes care (control group) or usual care plus a nurse-led diabetes self-management education (intervention group). Duration of the intervention was 12 weeks. The primary outcome was glycosylated hemoglobin (HbA1c values). Secondary outcomes were changes in blood pressure, body weight, lipid profiles, self-efficacy (efficacy expectation and outcome expectation), self-management behaviors, quality of life, social support, and depression. Outcome measures were assessed at baseline and at 12-week and 24-week postrandomizations. Patients in the intervention group showed significant improvement in HbA1c, blood pressure, body weight, efficacy expectation, outcome expectation, and diabetes self-management behaviors. The beneficial effect of a nurse-led intervention continued to accrue beyond the end of the trial resulting in sustained improvements in clinical, lifestyle, and psychosocial outcomes. This trial is registered with IRCT2016062528627N1.

## 1. Introduction

Iran is located in western Asia covering a total land area of 1,648,195 km^2^. By 2014, with a population of 82,801,633, Iran became the 16th most populous country in the world [1]. Healthcare in Iran is the responsibilities of the Ministry of Health (MOH) [2]. The national health priorities include developing a new policy to improve health system and better health outcomes for both communicable and noncommunicable diseases [3]. One of the great challenges that healthcare continues to face is the epidemiological transition of diseases from communicable to noncommunicable [4, 5]. Diabetes is a prominent public health concern in Iran that is associated with increased premature mortality, as well as increased risk for micro- and macrovascular complications. The International Diabetes Federation estimated that there were more than 4.6 million cases of diabetes in Iran in 2015, which is 8.5% of the population (20–79 years) [6]. The prevalence of diabetes in Iran is projected to be 9.2 million by the year 2040 [7]. Type 2 diabetes mellitus (T2DM) is the most common form of diabetes, accounting for 90–95% of all diabetes cases [8]. In Iran, T2DM occurs predominantly in middle-aged and older population, with an average age of 40–50 years. In contrast, the average age of the T2DM population in developed countries is over 65 years [9]. With the prolongation of the average life expectancy, the occurrence of T2DM in the younger age groups will lead to the escalation of the disease-related disability-adjusted life years in Iran [10]. In order to prevent or delay the development of diabetes complications, intensive efforts are required to achieve optimal glycemic control. Evidence suggested that diabetes self-management education can reduce diabetes complications and improve short-term glycemic control [11].

Different members of the healthcare team can provide diabetes self-management education. Much of research comparing the effectiveness of discipline-based education has not determined clear differences in the quality of services delivered by different healthcare professions [12]. However, published evidence favors the registered nurses, pharmacist, and registered nutritionist serving both as the members of the multidisciplinary team responsible for designing the curriculum and assisting in the delivery of education and the key primary instructors for diabetes education [12]. Nurses comprise the largest and most trusted health professional group. Nurses are uniquely positioned to inspire positive changes and transform healthcare delivery by serving as the bridge between theory and practice [13]. In diabetes self-management education (DSME), the close involvement of patients and caregivers is encouraged. Practice nurses are ideally positioned to provide monitoring, tailored feedback, and education on key aspects of self-management [14]. Despite growing interest, there is limited evidence to support the clinical effectiveness of nurse-led diabetes self-management interventions on glycemic control, particularly in Iran. The current study seeks to address these limitations by designing a randomized controlled trial with outcomes measured at baseline and postintervention at 3 and 6 months. Our primary hypothesis was that a nurse-led DSME intervention, compared with the usual care, would result in improvement in HbA1c. The secondary hypothesis was that the nurse-led DSME intervention would improve lipid profiles, blood pressure, body weight, self-management behavior, self-efficacy, quality of life, depression, and social support.

## 2. Method

### 2.1. Research Setting

This study took place in an urban primary and secondary outpatient endocrine clinic located within a teaching hospital in Ilam city, Iran. The hospital has a daily outpatient diabetes clinic with a daily turnover of approximately 20–30 patients except on Friday [15]. A multidisciplinary team is involved in the management of the patients. The clinic is currently the only leading medical service available to Ilamian residents with T2DM.

### 2.2. Research Design and Participants

The trial was conducted as a single-center, observer-blinded, parallel group (2 groups) randomized controlled trial.

Eligible participants were randomized into two groups:

1. The control group receives usual diabetes care routines.
2. The intervention group receives usual diabetes care plus 24 weeks of the nurse-led DSME intervention.

Participants were eligible if they were Iranian adults aged ≥18 years, who were clinically diagnosed with T2DM for at least 6 months, and had the medical record showing HbA1c ≥ 8%. To make sure that the lack of access to healthcare does not pose significant barriers to diabetes self-management, all patients were recommended to participate in follow-up at regular intervals. We required patients to participate in follow-up care (at least two visits per year) and have no evidence of a serious medical illness.

Exclusion criteria include cognitive dysfunction, pregnancy, uncontrolled high blood pressure (≥180/110 mmHg), hearing impairment, vision impairment, hemolytic anemias, and hemoglobinopathies. Patients were excluded if they were illiterate, had acute or chronic diabetes complications, and had major difficulties in activities of daily living.

The recruitment process was conducted in two stages. The first stage involved strategies to ensure adequate participant enrolment via placing an advertisement on the message boards of the clinic.

The second stage involved screening and assessment process by evaluating previous medical histories, measurement of HbA1c values, blood pressure, and visual acuity, and completion of three validated questionnaires regarding cognitive function (modified Mini-Mental State Examination (3MS)) [16], activity of daily living (Instrumental Activity of Daily Living (IADL)) [17], and hearing problem status (validated Single Global Screening Question) [18]. After successfully completing all screening processes and baseline assessment, eligible participants were randomized to either intervention or control groups. Randomization consisted of permuted block randomization with fixed block sizes of 4 or 6. No stratification was used. A statistician blinded to the allocation of participants to groups generates the random allocation sequence, using computer-generated random numbers. The allocation was concealed from the other researchers until randomization occurred.

In this study, we had multiple outcome variables, and the mean values were used for further analysis. Estimated sample sizes for each outcome variable were calculated independently, and the largest of the sample sizes was chosen as the target sample size. The following formula was used to calculate the sample size (Lemeshow et al., 1990: cited in [19]):

(1) $$\begin{array}{c} N=\frac{2\;\sigma^{2}{\left(Z_{1-\alpha /2}+Z_{1-\beta }\right)}^{2}}{{\left(\mu_{1}-\mu_{2}\right)}^{2}}. \end{array}$$

To detect the difference in means of outcomes, a minimum of 71 patients were needed for each arm (accounting for 20% loss to follow-up) [20].

### 2.3. Usual Care

The usual diabetes care is based on the Iranian Ministry of Health Guideline on the management of the T2DM, which involves self-care management, lifestyle modification, and medication adherence. Individual-based education was provided at three monthly intervals with the duration of 20–30 minutes per appointment. Face to face consultations and pamphlets are used to provide the usual diabetes care education. Physical examination and laboratory tests are performed at each visit in accordance with national guidelines. Based on the physical examination and laboratory findings, prescription refills or renewal are obtained during the check-ups.

### 2.4. Research Intervention

In addition to usual diabetes care, participants in the intervention group received a 12-week nurse-led DSME founded in the theoretical framework from Albert Bandura's self-efficacy theory [21] and Motivational Interviewing (MI) spirit. Self-efficacy was defined as “people's judgment of their capabilities to organize and execute the course of action which require designated types of performances” [22]. MI was used as a teaching approach toward the goal of behavior change [23, 24]. The American Association of Diabetes Educators (AADE) defined the seven essential self-care behaviors for successful and effective diabetes self-management. Self-care behaviors, including healthy eating, being active, monitoring, taking medication, problem-solving, reducing risk, and healthy coping, are the core components of our intervention.

Participants in the intervention group received (1) a detailed information booklet, which includes information on conducting self-management, (2) viewed four 10-minute movie clips, (3) attended four weekly group-based educational sessions, and (4) received follow-up telephone calls weekly.

A multidisciplinary team including endocrinologists, nutritionists, nurses, and pharmacists, with the actual intervention to be carried out by a nurse, designed the intervention program. The pilot version of the intervention was then produced and validated by fourteen patients with T2DM.

#### 2.4.1. Receiving the Booklet

The booklet consisted of six sections: (1) diet, (2) physical activity, (3) medication, (4) monitoring of blood glucose, (5) foot care, and (6) healthy living with diabetes. The booklet was developed based on the two sources of self-efficacy, that is, verbal persuasion and performance accomplishment. Participants in the intervention group received the booklet at the start of the program. The content of the booklet was used throughout the entire length of the intervention to direct further learning, elaboration, and discussion or to create resources for self-directed learning.

#### 2.4.2. Watching Movie Clips

For the first 4 weeks of the intervention, participants were invited to watch four 10-minute weekly movies. The movie is based on the content of the booklet. The language used in the movies was Persian. They were designed and developed by the research team to provide coaching and encouragement (verbal persuasion). Based on the educational contents, four movies were produced in different field of diabetes self-management including general information about T2DM, preventing short- and long-term complications (session 1), physical activity, daily foot care (session 2), healthy eating (session 3), and healthy living with diabetes (session 4).

#### 2.4.3. Group Discussion Session

Four group discussion sessions were carried out at weekly intervals, but the scheduling was flexible and the seating was limited to 10 participants for each session. These sessions were carried out weekly during the first 4 weeks of the intervention. Each session lasted for 120 minutes. The focus of these sessions was on building knowledge, self-efficacy, and skills regarding self-goal setting, action plan, problem-solving, sharing, and peer support. The group discussion sessions were facilitated by the first author (GA), who is a diabetes specialist nurse with 4 years of clinical experience. Participants' family members were encouraged to attend sessions that can provide multibeneficial support for the patient and their families. All group members were heartened to actively participate in each session. Group members who miss a session received a follow-up from the facilitator before the next session. Standard guidelines were developed for the group discussions and used in all the sessions held. At the last meeting of the group session, all group members received a list of residential phone numbers and were encouraged to ask for help if needed.

#### 2.4.4. Receiving Telephone Follow-Up Calls

Two months after the end of the group discussion sessions, intervention participants received a phone call once per week. Each follow-up call lasted approximately 15–20 minutes. The purpose of the telephone calls was to foster continued performance accomplishment via positive verbal persuasion. Telephone calls were based on principles of motivational interviewing (MI) that successfully help patients to engage and support them in making better health choices [25]. The first step in MI is to set the agenda for the consultation together with the patients [23]. “Agenda setting” is an issue to keep in the back of patient's mind from the opening scene of the interview. The basic question was “what are we going to talk about today?” Patients were encouraged to choose one or more key items (agenda settings) as their main area of concern. Then, the researcher assessed the participant's current self-care behavior and motivation for behavior change by rating and exploring importance and confidence with respect to the chosen key items. Techniques that were applied in the telephone calls to invoke the spirit of MI were asking open-ended questions, affirmations, reflective listening, summarizing during a conversation, expressing empathy, developing discrepancy, rolling with resistance, supporting self-efficacy, and reinforcing positive change-talk and new behavior. It led to an action plan being drawn up to implement strategies to improve the patient's performance [26]. At the end of the intervention, educational booklet and movies were given to the participants of the control group.

### 2.5. Measurements

#### 2.5.1. Laboratory Measures

A single laboratory analyzed all blood samples. For the blood tests, 10 cc of fasting venous blood was taken after fasting for at least 8–10 hours.

Whole blood levels of HbA1c, total triglyceride, total cholesterol, high-density lipoprotein (HDL), and low-density lipoprotein (LDL) were measured. NycoCard HbA1c analyzer (made in the US) was used for quantitative determination of HbA1c value. Lipid profile tests were conducted using an Auto Analyzer BT-3000 (made in Spain).

#### 2.5.2. Clinical Measures

Blood pressure was measured as the mean of two measurements performed after 5 min of rest while patients were seated with a cuff placed on their dominant arm at the same vertical height as the heart, using an automated blood pressure monitor (UA-779, A&D Instruments Ltd., Abingdon, UK). Weight and height were measured using a weighing and height scale (BT-ETS002, Medical Hospital Electronic height scale with weight measure, China). The participant's weight and height were measured without shoes or heavy clothing according to a standardized procedure. Patients were asked to stand up as straight as possible and look straight ahead. Body mass index (BMI) was calculated with the formula weight(kg)/height(m)^2^. According to the World Health Organization (WHO) criteria, there are three types of metabolic phenotypes as determined by BMI range (kg/m^2^) including underweight (severe thinness (<16), moderate thinness (16-17), and mild thinness (1–18.5)), normal BMI (18.5–25), and obesity (overweight (25–30), obese class I (30–35), obese class II (35–40), and obese class III (>40)) [27].

#### 2.5.3. Questionnaires

Efficacy expectation (self-efficacy) was measured using the Diabetes Management Self-Efficacy Scale (DMSES) [28]. The DMSES is a 20-item self-administered scale that assesses the extent to which respondents are confident that they can manage their blood glucose level, diet, level of physical activity, medications, and food care. The total score ranged from 0 to 200 points, with the higher scores indicating a greater self-efficacy [28]. This instrument has been validated for Iranian population [29] and showed to have satisfactory validity and reliability (*α* = 0.96).

Outcome expectation was measured using the Perceived Therapeutic Efficacy Scale (PTES) [30] translated into a Persian version in accordance with recommendations [31]. The PTES contains ten questions designed to measure the confidence that the individuals have in performing self-management which activates for achieving desired outcomes. The total score ranged from 0 to 100 points, with the higher scores indicating a greater confidence. The reliability of the Persian version of the instrument was 0.95. To date, this questionnaire had not been validated in Iran. Thus, a pilot study was conducted where the instrument was tested and validated at a hospital outpatient clinic in Ilam city before the questionnaire was taken into use. The pilot study was conducted in September 2016 among 160 individuals with T2DM who registered at the clinic. Pilot testing demonstrated that the instrument is valid and reliable to assess outcome expectation (*α* = 0.95).

Diabetes self-management behavior was measured using the validated Diabetes Self-Management Questionnaire (DSMQ). The instrument was translated from English into Persian in accordance with a recommended translation procedure [31]. The DSMQ is a 16-item self-administered questionnaire, with the higher scores indicating a more effective self-care [32]. To date, the DSMQ had not been validated for local use. Prior to the distribution of the instrument, a pilot study was conducted on 160 patients with T2DM to check the validity and reliability of the instrument. The pilot study indicated that the DSMQ is a valid and reliable instrument for assessing outcome expectations in Iran (*α* = 0.87).

The quality of life was measured using the World Health Organization Quality of Life Scale (WHOQOL-BREF). The WHOQOL-BREF is a self-administered, abbreviated version of the WHOQOL-100 containing 26 items divided into four domains and two general items. The WHOQOL-BREF scores were transformed on a scale from 0 to 100 with the higher scores indicating better quality of life [33]. This instrument has previously been validated for use in Iranian population (*α* = 0.94) [34].

Social support was measured with the Medical Outcome Study (MOS) Social Support Survey (SSS) tool. The MOS is a 19-item self-report instrument, measuring a multidimensional of the functional aspects of perceived social support, developed for patients with chronic conditions [35]. The total score ranged from 0 to 100 points, with the higher scores indicating more available social support. This instrument has previously been validated for use in Iranian population (*α* = 0.97) [36].

Depression was assessed using the Centre for Epidemiology Studies Short Depression Scale (CES-D) [37]. The CES-D is a 10-item self-administered instrument designed to measure the presence of depressive symptoms over the previous week. The total score ranged from 0 to 30 points, with a higher score indicating a more severe depression. This instrument has previously been validated for use in Iranian population (*α* = 0.93) [38].

### 2.6. Statistical Analysis

All data were analyzed using SPSS software (version 22, IBM Company, Chicago, IL, USA). Statistical significance was reported at the 0.05 alpha level, with two-tailed *P* values. Values are expressed as mean ± SD or *n* (%). Data analysis was carried out in two steps. Firstly, comparisons of baseline data between two experimental groups were made using Student's *t*-test or Mann–Whitney *U* test for continuous variables based on their normality, and the Chi-square or Fisher exact test for categorical variables, as appropriate. Next, main analyses were made on an intention to treat basis using repeated measures ANOVA. We investigated the impact of missing data with an expected-maximization algorithm, to compute estimates to replace missing data. Time points were used as the within-subject factor and group as the between-subject factor. Effect size is reported as partial Eta square. Our findings only concern the interaction of time × group.

### 2.7. Research Ethics

Ethical approval was granted from the University Putra Malaysia Ethics Committee for Research Involving Human Subjects and Medical University of Ilam Ethics Committee. Patients were informed about the purpose of the study and gave their consent prior to participation.

## 3. Results

### 3.1. Baseline Data

Between October 2016 and Jun 2017, 348 patients were approached and invited to participate in this study. Eighty-three patients did not meet the study inclusion criteria, and 78 declined to participate. The main reasons for declining participation were time constraints and family obligation. Of the 187 eligible patients, 13 did not respond (either telephone contact was not achieved after 6 repeated attempts or wrong phone number), 12 agreed to attend but did not, 11 not interested after reading the information, 7 responded after the deadline, and 2 not willing to be randomized. In total, 142 eligible patients were randomized; 72 were allocated to the intervention group and 72 were allocated to the control group.

**Figure 1:**
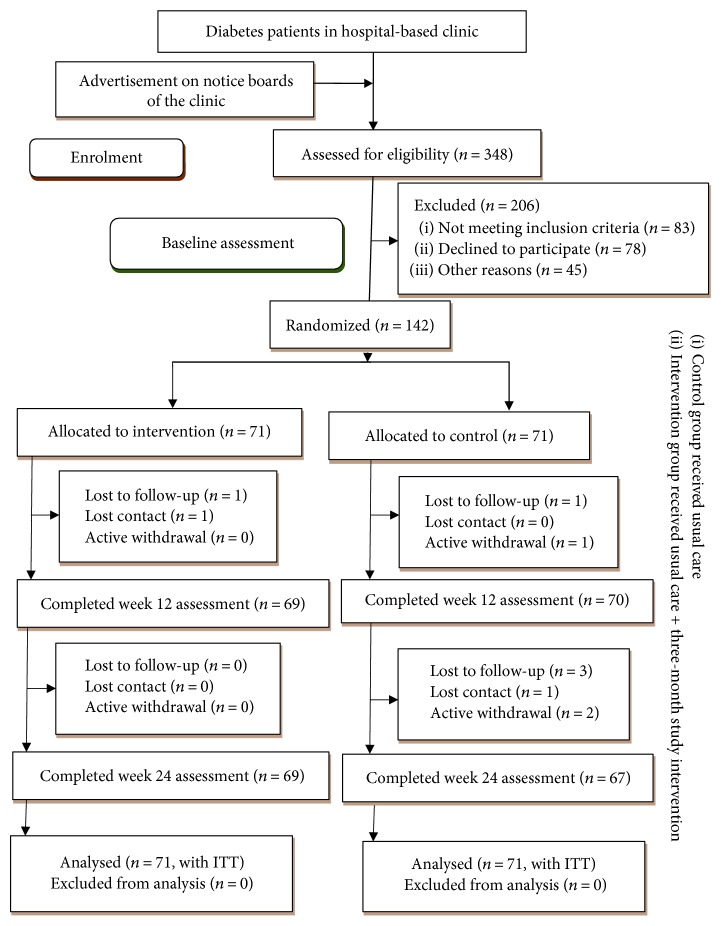
Consort flow diagram of study participation.

During the 24-week follow-up period, 6 patients dropped out (4.2%), of whom 5 had been allocated to the control group and 1 to the intervention group (Figure 1). The baseline characteristics of both groups were similar, indicating that randomization was effective. The differences between participants who completed and those who did not complete the study were not significant except for income. Income is a well-known factor that influences health outcomes. The analysis found that lower income individuals were more likely to withdraw from the study (*P* < 0.001).

Mean age at baseline was 54.2 ± 11.8 years (range 22–69 years), and two-thirds of the participants were female (65.5%). Majority of the participants had primary education (45.1%), and nearly three-fifths of them (58.5%) were currently working. A vast majority of participants had middle socioeconomic status; only 4.9% reported having difficulties meeting basic needs. Approximately three-quarters of the participants (76.1%) never smoked. The mean duration of diabetes was 8.9 ± 7.4 years. Participants were characterized by poor glycemic and blood pressure control. 58.5% had HbA1c ≥ 9%, and 9.1% had blood pressure at the recommended target < 130/80 mmHg. The proportion of self-blood glucose monitoring was similar for those who conducted it and those who did not, at 50% (see Table 1).

### 3.2. Laboratory Measures

The intention-to-treat analysis evaluated the primary and secondary outcomes. The primary outcome of the present study was HbA1c levels as presented in Table 2. Analysis of variance with repeated measure on time revealed a significant group-by-time interaction for HbA1c levels (*P* < 0.001). At week 12, participants in the intervention group had significantly lower HbA1c values (47.9%) than those in the control group. At week 24, the differences increased to 62% (*P* < 0.001). More than a fifth (21.1%) of the patients in the intervention group achieved an HbA1c < 7% compared to none in the control group (*P* < 0.001). No significant group × time interaction effects were observed for any of the lipid profile parameters except for the triglyceride.

### 3.3. Clinical Measures

Two-way ANOVA with repeated measures over time revealed statistically significant differences in the changes in systolic blood pressure and diastolic blood pressure from baseline at time points (12 and 24 weeks) between the two groups (*P* < 0.001). A significant interaction effect of time and group exists with respect to the body weight of participants (*P* < 0.001).

### 3.4. Questionnaires

Analysis indicated a significant group-by-time interaction for efficacy expectation, outcome expectation, and diabetes self-management behavior (*P* < 0.001). Mean scores for efficacy expectation, outcome expectation, and self-management behavior differ significantly between groups at postintervention, with the intervention group showing greater improvement compared with the control group.

Interestingly, our findings showed a significant interaction effect of time × group for quality of life (*P* < 0.001), but no significant group (*P* = 0.92) or time main effects (*P* = 0.86). There was a significant difference in social support scores at all time points in both the control and intervention groups (*P* < 0.001).

No statistically significant interaction effect of time × group in depression was found in our study (*P* = 0.10), although a small reduction in depressive symptoms was more likely in the intervention participants (mean differences at 6 months = −0.2, *P* > 0.05) than in the control group (mean differences at 6 months = −0.04, *P* > 0.05). No serious adverse events or withdrawals as a result of adverse events occurred in our study.

## 4. Discussion

We tested the hypothesis that nurse-led DSME program is effective in improving lifestyle, clinical, and psychosocial outcomes. Participants in the intervention group showed significant improvements in glycemic control, blood pressure, body weight, efficacy expectation, outcome expectation, self-management behaviors, and social support compared with patients in the control group. These improvements were sustained over a 24-week follow-up period.

No adverse effects of our intervention were reported by our subjects. In addition, none of the patients were hospitalized or died for hypoglycemia events. These findings indicate the patient's acceptance of the intervention as well as the success of the logistics. Further multicenter studies with larger sample sizes are needed to strengthen our results.

We had a dropout rate of 5% in this 24-week follow-up study which is statistically considered to an acceptable figure. This result suggested that the intervention procedures were well tolerated and there were no complications. However, dropouts were more likely to occur in low or lower-middle income individuals. Financial problems are known to significantly affect diabetes self-management in varying ways [39]. Individuals with low income face more environmental risk factors associated with poor diabetes control, such as lack of access to healthy foods, difficulties in accessing and paying for healthcare, the high costs of medical equipment, lack of access to tailored physical activity programs, and the stress and isolation. Growing evidence suggests that tracking broader issues of poverty is the key to better diabetes management [40].

The compliance and satisfaction of patients with the program were generally high, so the feasibility of implementing this intervention in a future trial on a broader scale is promising. The total cost of implementing the study intervention was $5000.

Unlike many previous studies that examined drug treatment effect on glycemic control, we employed a nurse-led DSME to improve self-management behaviors. Most of the antidiabetes drugs have serious adverse effects that led researchers to the selection of alternative strategies aiming at achieving a better control of diabetes. A recent controlled study suggests that a nurse-led DSME can achieve a greater decrease in HbA1c at 6 months [41]. In line with the above findings, our intervention found to be effective in decreasing glycated hemoglobin levels over 24 weeks, comparable to those of drug trials. Overweight individuals with type 2 diabetes are two to four times more likely to develop cardiovascular complications. In fact, a small amount of weight loss (≥2%) in diabetic patients seems to mediate significant improvements in cardiovascular risk factors [42]. The extent of body weight loss was significantly larger in the intervention group compared to the control group (−0.58 ± 0.09 versus 0.07 ± 0.08, *P* < 0.05). Although the mean reduction in body weight was modest (0.58 kg decrease), one should not underestimate the potential benefits of weight loss and improvement in cardiovascular risk factors. The intervention group showed statistically significant improvements in blood pressure levels compared to the control group. Overall, our intervention resulted in a modest but significant reduction in weight, which, in turn, leads to significant decrease in HbA1c and blood pressure. There are numerous factors that can lead to changes in these variables, but lifestyle modifications is the key factor in the management of diabetes. Changes in lifestyle and dietary habits may have led to weight reduction over time. Losing weight can help control blood glucose levels and reduce blood pressure [43, 44]. The current study is in agreement with previous studies, demonstrating that modest weight loss can mitigate cardiovascular risk factors [11].

There were no significant differences between groups in their lipid profiles. However, there was a nonsignificant tendency towards a greater improvement in lipid profiles that might be confirmed if we had the bigger sample size and longer follow-up period. Recent studies suggest that lipid profile values varied by season, tend to be worse in cold season than in the warm season [45]. This study was conducted from October to May when the average air temperature decreased over the study period. We hypothesize that seasonal variation may be related to the changes in this parameter rather than the outcome of our intervention.

This study was based on a theoretical construct of self-efficacy by Bandura, the perception of one's ability to perform a task successfully [46]. This study has taken an initial step in involving nurses to work and collaborate beyond the traditional boundaries in order to build on the self-efficacy. This might be the key attributes for increasing patients' skills and confidence in managing health problems—so-called “self-management program” instead of just “self-monitoring” which has been proven to be not very useful. Our results were generally in accordance with the theoretical construction of the self-efficacy. Participants in the intervention group had significantly greater improvements in efficacy expectation, outcome expectation, and self-management behaviors compared with the control group. These results provide further support to drive a paradigm shift in healthcare delivery system form doctor-centered to patient-centered approaches. This potentially puts more emphasis on supporting nursing education, patients' self-management, and confidence to accomplish activities rather than mainly hospital-based management.

Our findings showed a significant interaction effect of time × group for quality of life (*P* < 0.001), but no significant group (*P* = 0.92) or time main effects (*P* = 0.86). One possible explanation for this finding is that there is a crossover interaction [47]. The existence of crossover interaction suggests that at 3-month intervention group showed better quality of life than the control group, but that this pattern reversed for the 6-month measurement. Although nonsignificant, our intervention had a positive impact on how patients perceived their quality of life.

Social support is known as one of the emotional coping mechanisms that can positively influence the quality of life [48]. There is a direct positive relationship between social support and health, so higher levels of social support are linked to better overall health [49, 50].

We found a significant improvement in social support throughout the study period. In contrast, no significant differences between groups were observed for quality of life and depression, which likely reflect the short follow-up period and lack of power to detect such changes. However, there was a nonsignificant trend towards greater improvements in quality of life and depression symptoms in the intervention group than in the control group that might be confirmed if we had the larger sample size and longer follow-up period. To substantiate the robustness of the findings, further detailed interventions with larger sample size and longer follow-up period are still required to confirm our results.

### 4.1. Strength and Limitation

To the best of our knowledge, this is the first study of its kind conducted in Ilam province, in the western part of Iran.

This trial has many strengths. By using a randomized clinical trial, we ensured a robust study design, with reasonably well-matched pairs in groups. Eventually, we were successful in minimizing contamination between the two groups. The program was delivered by a facilitator trained to give a high-quality education program. We aimed to ensure that necessary education materials were available to all in a local language. This approach greatly increases the generalizability of our findings and therefore the possibilities to implement this program for use in other resource-limited settings.

Data were analyzed by the intention-to-treat approach to preserve the merit of randomization. At the end of the study, the response rate was higher than expected (more than 90%), minimizing the amount of missing data. We acknowledge that there are some limitations in our study. Participants were followed-up for a short period of time. A follow-up period of 6 months may be too short to evaluate the long-term effect of the self-management intervention. Further studies are needed to evaluate the long-term effects of the intervention. Our trial may have been underpowered to detect significant changes in some variables and as a result, some of our findings may be prone to type 2 error (i.e., discarding real associations). There was a substantially and statistically significantly greater improvement in the intervention group, which might be due to the Hawthorne effect. The Hawthorne effect can influence the behavior of the participant, potentially improve or modify their behavior in response to the fact that they knew they are being observed. However, a rigorous design was used to minimize and adjust the common bias and limitations associated with research.

## 5. Conclusion

In a cohort of 142 adults with T2DM, a single-center nurse-led DSME offered sustained benefits in clinical and lifestyle outcomes at 24 weeks. Facilitating self-efficacy has been found to improve longer-term health outcomes in patients with chronic health conditions [51]. Our findings indicated that it is possible to achieve behavior changes by enhancing intrinsic motivation and self-efficacy. Thus, the novelty here is the application of theory into the clinical practice. Our findings support the implementation of a program that emphasizes collaborative learning, although the optimum interval and contact time require further evaluation. We recognize that there might be room for further improvement of clinical outcomes by increasing contact time and frequency. Further research, with extended contact time and longer follow-up, is needed to show if our intervention has long-term effects.

## Figures and Tables

**Table 1:** Sociodemographic characteristics of study participants.

Characteristics	Total sample *N* = 142	Intervention group *N* = 71	Control group *N* = 71
Age, years^a^	56 ± 11.1	55.09 ± 10.16	53.49 ± 10.98
Gender^b^			
(i) Male	49 (34%)	23 (32.4%)	26 (36.6%)
(ii) Female	93 (65.5%)	48 (67.6%)	45 (63.4%)
Marital status^b^			
(i) Married	117 (82.4%)	58 (81.7%)	59 (83.1%)
(ii) Single (divorced/widow)	25 (17.6%)	13 (18.3%)	12 (16.9%)
Educational status^b^			
(i) Primary education	64 (45.1%)	33 (46.5%)	31 (43.7%)
(ii) Secondary education	23 (16.2%)	12 (16.9%)	11 (15.5%)
(iii) Tertiary education	55 (38.7%)	26 (36.6%)	29 (40.8%)
Occupation status^b^			
(i) Working	83 (58.5%)	28 (39.4%)	31 (43.7%)
(ii) Not working	59 (41.5%)	43 (60.6%)	40 (56.3%)
Difficulty paying for basics^b^			
(i) Very hard	7 (4.9%)	5 (7%)	2 (2.8%)
(ii) Somewhat hard	96 (67.6%)	47 (66.2%)	49 (69%)
(iii) Not hard at all	39 (27.5%)	19 (26.8%)	20 (28.2%)
Smoking status^b^			
(i) Current smoker	18 (12.7%)	9 (12.7%)	9 (12.7%)
(ii) Never	108 (76.1%)	55 (77.5%)	53 (74.6%)
(iii) Ex-smoker	16 (11.3%)	7 (9.9%)	9 (12.7%)
Duration of diabetes, years^a^	8.9 ± 7.4	8.8 ± 7.5	9.04 ± 7.31
Presence of at least one comorbidity^b^	113 (79.5%)	57 (80.3%)	56 (78.9%)
Use of SBGM^a^	71 (50%)	32 (45.1%)	39 (54.9%)
BMI (kg/m^2^)^a^	28.78 ± 3.34	28.69 ± 3.25	28.87 ± 3.46
Systolic blood pressure (mmHg)^a^	132.3 ± 11.2	130.6 ± 9.6	133.9 ± 12.4
Diastolic blood pressure (mmHg)^a^	86.6 ± 5.9	85.8 ± 5.2	87.3 ± 6.4
HbA1c^a^	9.32 ± 1.11	9.32 ± 1.06	9.31 ± 1.15
(i) 8–8.9%^b^	59 (41.5%)	29 (40.8%)	30 (42.3%)
(ii) ≥9%^b^	83 (58.5%)	42 (59.2%)	41 (57.7%)
Triglyceride^a^	142.4 ± 34.9	142.83 ± 34.4	142.09 ± 35.7
Total cholesterol^a^	172.5 ± 46.8	173.14 ± 45.4	171.91 ± 48.4
HDL^a^	53.44 ± 12.62	53.68 ± 12.8	53.21 ± 12.4
LDL^a^	94.28 ± 27.86	95.89 ± 30	92.68 ± 25.6

Note: ^a^Mean ± standard deviation; ^b^frequency (%); HDL = high-density lipoprotein; LDL = low-density lipoprotein; BMI = body mass index; SBMG = self-blood glucose monitoring.

**Table 2:** Two-way ANOVA with repeated measures for primary and secondary outcomes.

Model	Intervention mean (SD)			Control mean (SD)			Significance		
	*T*_1_	*T*_2_	*T*_3_	*T*_1_	*T*_2_	*T*_3_	*F* value	*P* value	Effect size (Eta square)
*HbA1c*	9.3 (1.06)	8.6 (1.01)	7.9 (0.93)	9.3 (1.1)	9.3 (1.1)	9.3 (1.1)			
Group							14.5	*P* < 0.001	0.09
Time							277.6	*P* < 0.001	0.66
Group × time							289.5	*P* < 0.001	0.67
*Systolic blood pressure*	130.7 (9.4)	129.3 (9.1)	127.8 (9)	133.9 (12.3)	133.7 (12.1)	133.3 (12)			
Group							5.9	0.01	0.04
Time							59.1	*P* < 0.001	0.29
Group × time							25.7	*P* < 0.001	0.15
*Diastolic blood pressure*	85.8 (0.69)	84.7 (0.67)	83.4 (0.65)	87.3 (0.69)	87.3 (0.67)	87.1 (0.65)			
Group							7.4	*P* < 0.001	0.05
Time							61.3	*P* < 0.001	0.30
Group × time							39.3	*P* < 0.001	0.21
*Weight*	82.58 (11.08)	82.21 (11.09)	82 (10.89)	83.75 (10.93)	83.77 (10.91)	83.82 (10.89)			
Group							0.68	0.41	0.01
Time							4.69	0.01	0.03
Group × time							7.17	0.01	0.04
*BMI*	28.69 (3.2)	28.54 (3.2)	28.55 (3.2)	28.87 (3.4)	28.87 (3.4)	29.89 (3.4)			
Group							0.24	0.62	0.01
Time							2.59	0.07	0.01
Group × time							3.18	0.04	0.02
*Triglyceride*	142.83 (34.4)	141.66 (33.9)	140.90 (33.7)	142.09 (35.7)	142.04 (35.6)	142.01 (35.6)			
Group							0.00	0.96	0.01
Time							40.41	*P* < 0.001	0.22
Group × time							33.82	*P* < 0.001	0.19
*Cholesterol*	173.1 (45.4)	171.3 (43.8)	169.09 (43.1)	171.9 (48.4)	170.7 (47.02)	171.88 (46.8)			
Group							0.006	0.93	0.01
Time							3.15	0.06	0.02
Group × time							0.10	0.82	0.01
*LDL*	95.89 (30.04)	95.59 (28.4)	93.95 (27.9)	92.68 (25.6)	93.06 (25.4)	94.15 (25)			
Group							0.16	0.68	0.01
Time							0.15	0.85	0.01
Group × time							5.62	0.22	0.39
*HDL*	53.68 (12.8)	54.72 (11.6)	55.38 (11.8)	53.21 (12.4)	53.21 (12.5)	52.76 (12.6)			
Group							0.55	0.45	0.01
Time							2.66	0.28	0.01
Group × time							6.94	0.94	0.04
*Efficacy expectation*	98.35 (13.95)	113.47 (11.07)	123.47 (11.31)	98.06 (17.47)	99.77 (14.48)	98.44 (15.69)			
Group							34.3	*P* < 0.001	0.19
Time							152.2	*P* < 0.001	0.52
Group × time							138.7	*P* < 0.001	0.49
*Outcome expectation*	57.80 (7.11)	62.85 (5.50)	66.79 (5.35)	58.11 (7.50)	58.04 (7.33)	58.16 (7.23)			
Group							16.15	*P* < 0.001	0.10
Time							150.81	*P* < 0.001	0.51
Group × time							147.61	*P* < 0.001	0.51
*DSMB*	3.56 (1.22)	4.67 (1)	5.41 (1.15)	3.71 (1.40)	3.67 (1.37)	3.73 (1.40)			
Group							16.7	*P* < 0.001	0.10
Time							228.5	*P* < 0.001	0.62
Group × time							221.6	*P* < 0.001	0.61
*QOL*	50.42 (9.17)	50.76 (9.06)	50.67 (9.04)	50.69 (8.90)	50.34 (8.83)	50.39 (8.71)			
Group							0.00	0.92	0.01
Time							0.07	0.86	0.01
Group × time							10.5	*P* < 0.001	0.07
*Social support*	52.63 (9.31)	54.33 (9.42)	55.37 (9.61)	55.83 (12.11)	55.82 (12.63)	55.91 (12.75)			
Group							0.88	0.34	0.01
Time							43.6	*P* < 0.001	0.23
Group × time							39.3	*P* < 0.001	0.21
*Depression*	12.15 (4.99)	11.98 (4.97)	11.95 (5.03)	12.76 (4.66)	12.84 (4.61)	12.91 (4.50)			
Group							1	0.31	0.01
Time							0.51	0.55	0.01
Group × time							9.33	0.10	0.06

Note: Group: test of between-subject effects; Time: test of within-subject effect; Group × time: interaction between group by time; SD: standard deviation; *T*_1_: baseline measurement; *T*_2_: 3-month measurement; *T*_3_: 6-month measurement; DSMB: diabetes self-management behavior; LDL: low-density lipoprotein; HDL: high-density lipoprotein; BMI: body mass index; QOL: quality of life; Primary outcome: HbA1c. To interpret effect sizes, 0.02 be considered a “small magnitude,” 0.15 be considered a “medium magnitude,” and 0.35 be considered a “large magnitude” [52].
